# First Description of a New Disease in Rainbow Trout (*Oncorhynchus mykiss* (Walbaum)) Similar to Heart and Skeletal Muscle Inflammation (HSMI) and Detection of a Gene Sequence Related to Piscine Orthoreovirus (PRV)

**DOI:** 10.1371/journal.pone.0131638

**Published:** 2015-07-15

**Authors:** Anne Berit Olsen, Monika Hjortaas, Torstein Tengs, Hege Hellberg, Renate Johansen

**Affiliations:** 1 Norwegian Veterinary Institute, Bergen, Norway; 2 Norwegian Veterinary Institute, Oslo, Norway; INRA, FRANCE

## Abstract

In fall 2013, anorexia, lethargy and mortalities up to 10-12,000 dead fish per week were observed in rainbow trout *Oncorhynchus mykiss* in three fresh water hatcheries (salinity 0-1 ‰) on the west coast of Norway. The fish (25-100 g) showed signs of circulatory failure with haemorrhages, ascites and anaemia. The histopathological findings comprised inflammation of the heart and red muscle and liver necrosis. The affected fish had a common origin. Disease and mortalities were also observed up to four months after sea water transfer. Microbiological examination did not reveal presence of any known pathogens. Based on histopathological similarities to heart and skeletal inflammation (HSMI) in Atlantic salmon, associated with piscine orthoreovirus (PRV), extended investigations to detect a virus within the family *Reoviridae* were conducted. By the use of primer sets targeting the PRV genome, a sequence with 85% identity to a part of segment S1 of PRV was obtained. Further analysis showed that the virus sequence could only be aligned with PRV and no other reoviruses both on amino acid and nucleotide level. Two PCR assays were developed for specific detection of the virus. High amounts of the virus were detected in diseased fish at all affected farms and low amounts were detected in low prevalence at the broodfish farms. Further investigations are needed to determine if the virus is associated with the new disease in rainbow trout and to further characterize the virus with respect to classification, relationship with PRV, virulence, pathology and epidemiology.

## Introduction

Rainbow trout *Oncorhynchus mykiss* (Walbaum) is a North Pacific salmonid species which is farmed in many parts of the world. Rainbow trout is often farmed in fresh water dams and raceways. In Norway the main production is in sea locations. According to FAO-FishStat the global production of rainbow trout reached 850,000 tons in 2012, and is a growing industry. Norway produced approximately 71,000 tons rainbow trout in 2013 (Directory of Fisheries), which is a limited number compared to the production of Norwegian Atlantic salmon (*Salmo salar* L.) of more than 1 million tons.

Rainbow trout production may suffer serious losses due to infectious diseases, including viral diseases such as viral haemorrhagic septicaemia (VHS) and infectious haematopoietic necrosis (IHN). Bacterial diseases impose limitations on rainbow trout farming and infection with *Flavobacterium psychrophilum* is an important cause of mortality. In Norway, outbreaks of infectious pancreatic necrosis (IPN) in fresh water are reported. Some farms have experienced serious mortalities due to *F*. *psychrophilum* both in fresh water and in brackish sea locations [1]. At sea water locations the losses of rainbow trout due to infectious disease problems are sparse compared to Atlantic salmon, even though eight outbreaks of pancreas disease (PD) were recorded on rainbow trout in Norway in 2013 [2].

Heart inflammation, as described in the present paper, has not previously been observed in rainbow trout in Norway. Similar findings have though been published from Canada where rainbow trout was used in an experimental challenge with infectious salmon anaemia virus (ISAV) [3]. Immunohistochemical examination for ISAV did not reveal the cause of the heart inflammation and this particular pathology has not been observed in other ISA cases. Pancarditis is commonly seen in Atlantic salmon with the disease heart and skeletal muscle inflammation (HSMI) [4] which is associated with piscine orthoreovirus (PRV) [5], [6], [7]. HSMI in salmon is a problem mainly in sea water farms and anaemia is not described. Piscine orthoreovirus has been detected in other salmonids like rainbow trout, chum salmon (*Oncorhynchus keta*) and cutthroat trout (*O*. *clarkia*) in Canada, but the virus has not been associated with disease in these species [8].

The *Reoviridae* is a family of viruses that are non-enveloped, icosahedral shaped, with 9–12 segments of linear dsRNA and are found in a wide range of hosts including insects, plants, birds, mammals and fish. Most reoviruses from fish belong to the aquareovirus genus and their effects on fish health are in most cases not well documented [9]. Some strains have been isolated from diseased fish concurrently with other disease problems, and it is discussed whether reoviruses suppress the immune system, thereby making the fish more susceptible to other diseases, or if they are the main cause of the disease [10]. Recently a new aquareovirus was detected in Norwegian farmed Atlantic halibut fry (*Hippoglossus hippoglossus*) with high mortality rates [11] and this emphasises the need for more knowledge about the impact of these viruses on fish health. Diseases associated with reoviruses are to our knowledge previously not recorded from rainbow trout.

The aim of the present study was to describe a new disease in rainbow trout detected in three fresh water farms and to follow the surviving fish after sea transfer. A gene sequence was detected in diseased fish using a PRV-primer set, and two new PCR assays were established for specific detection. In the present study the new assays are used for mapping the distribution of the PRV-like virus in the affected farms and some of the contact farms including the two broodfish farms. The paper also includes an initial characterization of the virus. Further investigation is needed to fully characterize the virus and possibly show causality between the virus and the disease described in this paper.

## Materials and Methods

### Study material

**Fig 1:**
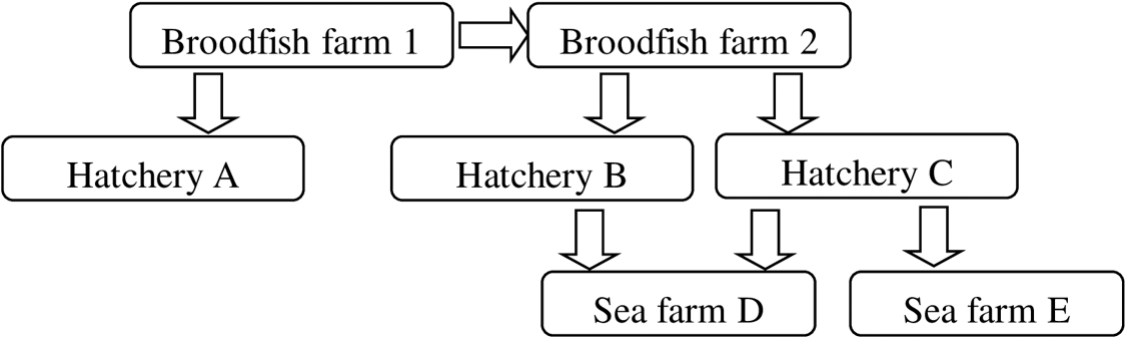
Overview of infected farms. Arrows show movement of eggs or fish. The disease was observed in all three fresh water hatcheries and the two sea farm locations. The PRV-related virus was detected in all seven farms.

Samples from seven farms are included in this study. Fig 1 provides a schematic overview of movement of eggs and fish between the farms. Fish and eggs from the broodfish and hatcheries described here have also been moved to other locations not included in this study. The first samples came from both diseased and clinically healthy fish at the three hatcheries A, B and C in the fall 2013. Later, samples were taken from clinically healthy fish at the broodfish farms. After transfer to sea in spring 2014 samples were provided from diseased and healthy fish at the sea farms D and E. Number of samplings per farm were three to five and total number of fish sampled per farm varied from 20 to 100. Before sampling the fish were killed with a blow to the head. Samples were delivered to the Norwegian Veterinary Institute, either as whole fish on ice or as tissue samples on formalin, RNA*later* or transport medium (Eagle’s Minimum Essential Medium with 10% newborn bovine serum and 100 μg ml^–1^ gentamicin). In some cases blood samples were received either on EDTA or heparin containers. From farm C heart samples were also fixed in a glutaraldehyde solution for electron microscopic examination.

### Histopathology

Gill, heart, liver, spleen, mid-kidney, pancreatic tissue, pyloric caeca and skeletal musculature from 113 fish were fixed in 10% neutral buffered formalin, embedded in paraffin and routinely processed. The sections, 3–4 μm, were stained by haematoxylin and eosin (H&E) and studied by light microscopy. A selected number of slides were also stained using Gram Twort, periodic acid Shiff (PAS), May Grünwald Giemsa (MGG) and Ziehl Neelsen (ZN).

### Electron microscopy

Heart tissues including blood cells from affected fish at farm C were fixed in a 1.25% glutaraldehyde and 2% formaldehyde PBS solution for a minimum of 24 h. Tissues were then washed several times in PBS and cacodylate buffer (0.1 M, pH 6.8) previous to post fixation with Osmium (OsO4) in 1 h and washed again in cacodylate buffer. Dehydration in an ethanol series (70, 90, 96, 100%) was then performed before embedding in LR-White resin (Electron Microscopy Sciences, USA) and ultra-sections were made. Sections were stained with 4% aqueous uranyl acetate and 1% potassium permanganate (KMnO4) for 10 min and then washed in distilled water before examined and photographed in the transmission electron microscope (TEM, FEI MORGAGNI 268).

### Bacteriology

Four to 20 samples from kidney of diseased fish from hatcheries A, B and C were inoculated onto blood agar as well as Anacker and Ordal’s medium (A&O) [12] and incubated at 22°C and 15°C respectively for 7 d.

### Immunohistochemistry

An additional method to cultivation on A&O was used to test if *Flavobacterium psychrophilum* could be associated with the lesions. A selection of heart, kidney and spleen tissue with lesions from 13 fish representing all three hatcheries were tested for *F*. *psychrophilum* antigen with the enzyme-labelled (alkaline phosphatase) streptavidin procedure, with fast red as the chromogen. A polyclonal rabbit antiserum raised against *F*. *psychrophilum* serovar Th was used [13].

### Virus cultivation

Heart, kidney and spleen tissues from 10 diseased fish from farm C were collected and homogenized in viral transport medium (TM) (Eagle’s Minimum Essential Medium with 10% newborn bovine serum and 100 μg/mL gentamicin). For virus cultivation, tissue homogenates were inoculated on Chinook salmon embryo (CHSE-214) cell monolayers [14], Bluegill fry-2 (BF-2) and epithelioma papillosum of carp (EPC) cell lines as described in Commission Decision 2001/183/EC http://eurlex.europa.eu/LexUriServ/LexUriServ.do?uri=OJ:L:2001:067:0065:0076:EN:PDF.

### Real time RT-PCR (rRT-PCR) for known viruses

Samples were screened by rRT-PCR for the detection of a range of RNA viruses capable of infecting salmonids. A minimum of three diseased fish from farms with clinical disease (farm A, B, C, D, E) were tested for piscine orthoreovirus (PRV), infectious salmon anaemia virus (ISAV), salmonid alphavirus (SAV), piscine myocarditis virus (PMCV), viral haemorrhagic septicaemia virus (VHSV), infectious hematopoietic necrosis virus (IHNV) and infectious pancreatic necrosis virus (IPNV) using primers and probe as described elsewhere [5], [15], [16], [17], [18], [19], [20].

### Identification of a gene sequence by RT-PCR and Sanger sequencing

Preliminary histopathological findings indicated resemblance to HSMI in Atlantic salmon. As HSMI is associated with PRV, investigations to detect a virus within the family *Reoviridae* were initiated. Total RNA from tissue samples from hatchery A was extracted using QIAsymphony SP (QIAGEN) in accordance with manufacturer’s instructions using the QIAsymphony RNA kit. Amplification of PCR products was performed by one-step RT-PCR using primer sets targeting parts of the PRV genome as described by Garseth and co-workers [21]. Reactions were conducted in a final volume of 20 μl with OneStep RT-PCR kit (QIAGEN). Five hundred ng total RNA and 0.75 μM of each primer was denatured (95°C/5 min) and mixed with 1x RT-PCR buffer, 400 μM dNTPs, 4 U RNaseOUT and 1 μl OneStep RT-PCR enzyme mix. The cycling parameters were as follows: cDNA synthesis 50°C/30 min followed by 95°C/15 min and PCR: 40 cycles of 94°C/30 s, 55°C/30 s and 72°C/60 s. The final PCR products were visualized by gel electrophoresis. All PCR products of approximately right size were sequenced using the amplification primers and the ABI Prism Big Dye Terminator Cycle sequencing kit on ABI Genetic Analyzer. Obtained sequences were assembled using the Sequencher 4.5 software from GeneCode and blasted against GenBank. While two of the three primer sets resulted in either no or unspecific amplifications, primers S1_39F and S1_621R provided a 562 bp nucleotide sequence displaying 85% identity to parts of S1 segment of PRV with accession number GU994022. This sequence (Acc. No. LN680851) was used to establish the RT-PCR assays described below. Alignment and phylogenetic analysis were performed in order to visualize the genetic relationship between the virus sequence detected in our study and other viruses using the software SeaView (v. 4.5.3) [22].

### Real time RT-PCR for detection of the PRV-related virus

Forward primer 5’- TCG-TGG-TTC-CAA-TGA-CAG-3’, reverse primer 5’- CCA-ACC-ACT-AAA-ACC-GAG- 3’ and probe 6-FAM-5’- ACG-CCT-TAG-AGA-CAA-CAT-GCG-AAG-3’- BHQ-1 were designed for amplification and detection of a 121 bp fragment of the genome of the PRV-like virus by rRT-PCR.

The rRT-PCR assay was performed using the QIAGEN OneStep kit. Reactions were conducted in a final volume of 20 μl with ROX passive reference dye RT-RARE_03 (Eurogentec) and RNaseOUT (Invitrogen). Five hundred ng of total RNA from fish displaying clinical signs of the new disease was mixed with 1x RT-PCR buffer, 30 nM ROX, 400 μM dNTPs, 0.5 μM of each primer, 0.3 μM probe, 1.25 mM MgCl_2_, 4 U RNaseOUT and 0.8 μl OneStep RT-PCR enzyme mix. Amplifications were done on a Stratagene Mx3005P real time machine (Stratagene) with following conditions: 30 min at 50°C (reverse transcription), followed by 15 min at 95°C (RT inactivation and activation of *Taq* polymerase), 40 cycles of 94°C/30 s, 55°C/30 s and 72°C/30 s. Specificity of the rRT-PCR assay was determined by blasting of the nucleotide sequences of forward and reverse primers and the probe against GenBank using BLAST X to identify any known organism with which they might cross-react. Additionally, the newly established rRT-PCR assay was tested against nucleic acid extracted from a selection of other RNA viruses capable of infecting salmonids including PRV, ISAV, SAV, PMCV, VHSV, IHNV and IPNV.

### Screening of the farms for the PRV-related virus

From each of the seven farms included in this study (Fig 1), 20–50 fish were analyzed with the newly established rRT-PCR method for detection of the PRV-like virus. In the first cases from 2013, blood and several tissues including heart and kidney were tested. As the initial screening results showed highest amount of virus in blood at the acute stage of the disease, mainly blood was tested from the subsequent cases.

Total nucleic acid from organ samples on RNA*later* was extracted as described elsewhere [23]. The following protocol was established for extraction of nucleic acid from blood: 100 μl of blood on EDTA or heparin was mixed with 750 μl of lysis buffer (BioMérieux) and homogenized using Mixer Mill MM 400 (Retsch). Total nucleic acid was extracted on automated NucliSENS easyMAG (BioMérieux Norge AS, Oslo, Norway) in accordance with the manufacturer's protocol for whole blood and on board lysis. Analyses were performed with QIAGEN OneStep kit and 500 ng nucleic acid using the rRT-PCR protocol for detection of the virus as described above.

### Conventional RT-PCR for verification

For verification of the positive rRT-PCR findings a conventional RT-PCR assay was designed. Primers 6F 5’-GAC-CAA-CAT-AAC-GTT-TCA-GGC-3’ and 6R 5’-ATC-CAA-CCA-CTA-AAA-CCG-AGA- 3’ amplifying a 423 bp fragment of the genome of the virus were applied together with the protocol for the conventional RT-PCR as described above omitting the RNA denaturation step. Positive findings from each site were confirmed by sequencing of the obtained products using the reverse primer from the rRT-PCR assay and primers 6F&6R.

### Statistical analysis

Unpaired Student’s *t*-test was used to compare means. The results are presented as mean ± SDs of means of the data.

### Ethics statement

This study meets all applicable standards for the ethics of research integrity. The fish samples were collected by fish health personal authorized to investigate fish disease and was part of their regular disease diagnostic activity. No endangered or protected species were involved.

## Results

### Epidemiology

Fig 1 shows the connection between the farms. The first disease outbreak was recorded in farm B in August 2013. Farm A and C were diagnosed with the same condition in October and November respectively. The fish, weight 25–100 g, were held in fresh water with no or minimal addition of sea water (<1 ‰). Water temperature at the start of clinical disease was 7–10°C. For farm A the disease was observed mainly in two tanks, mortalities were only moderately increased and no clinical disease has been observed after sea transfer, except for one group which experienced some mortalities the first few days after transfer. Farm B reported low to moderate mortalities in some tanks (0.1–1.5% per week), but at sea transfer in autumn 2013 high mortalities were observed during the first days in sea water. No clinical disease or mortalities have been recorded at this sea farm later on. Farm C experienced clinical disease in several tanks and mortalities were observed for approx. six months. Up to 10–12,000 fish died per week. Accumulated mortalities varied from 0.3% in tanks with apparently healthy individuals to 21% in a tank with diseased fish. In February-April 2014 surviving fish from affected groups in hatcheries B and C were transferred to sea farm D and from farm C also to sea farm E. In both sea farms the disease was diagnosed up to four months after sea transfer and total mortalities were 5.5% and 2.5% respectively, which also include mortalities due to unspecific causes.

### Clinical signs and gross pathology

Typical clinical signs were loss of appetite and lethargy. Fish seemed to stay along the wall of the tank and high in the water column. The lethargic stage could last for days before mortalities occurred.

**Fig 2:**
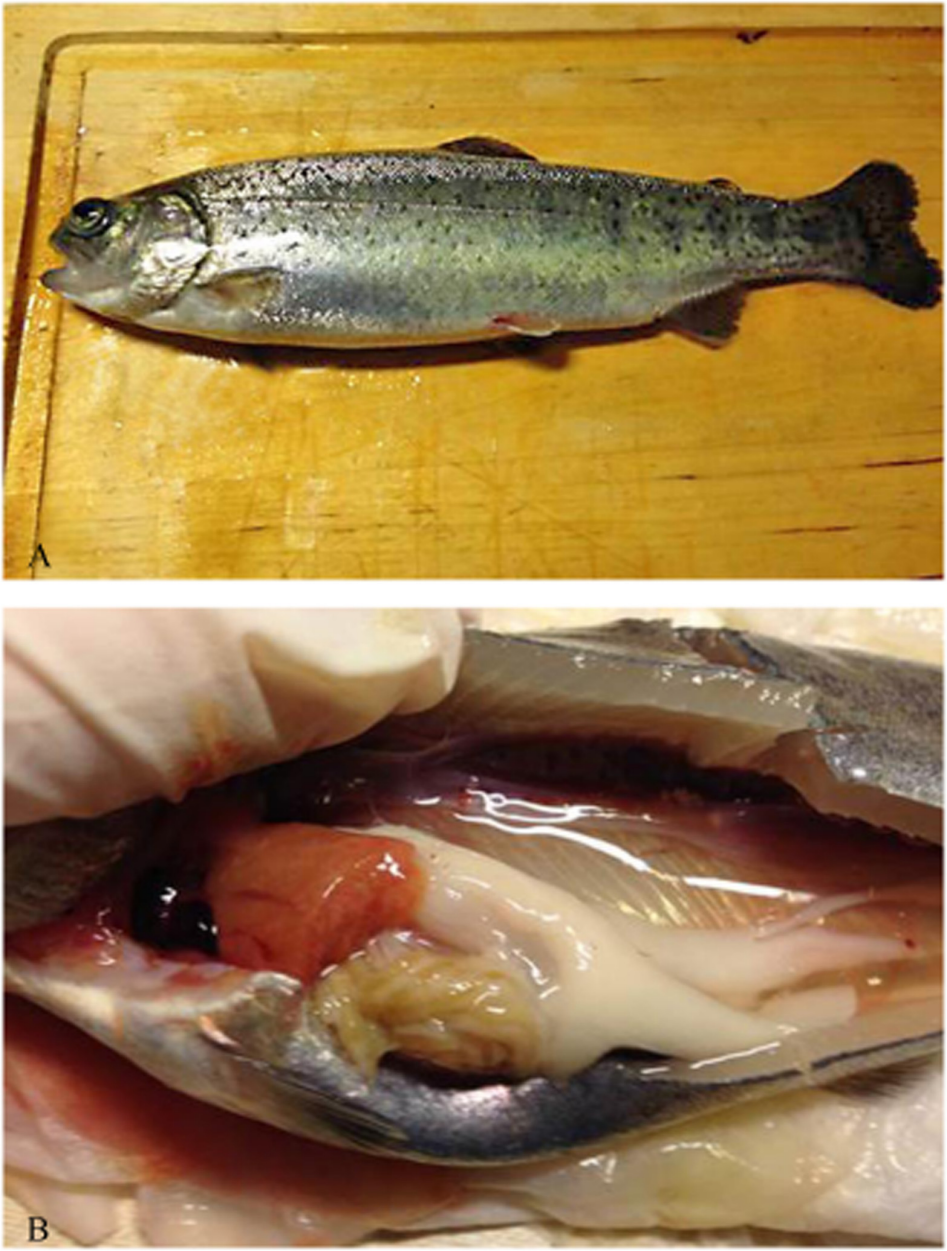
Diseased rainbow trout. (A) Sick fish with exophthalmos and haemorrhaged bases of fins. (B) Fish with haemopericardium due to haemorrhage of the heart, pale liver and serohaemorrhagic ascites.

The skin and gills were pale and bilateral exophthalmos was often observed (Fig 2A). Cutaneous haemorrhages, particularly on the abdomen and at the bases of the pectoral and abdominal fins, were frequent findings. Autopsy often revealed a pale heart and sometimes dilated, blood filled atrium. Haemopericardium due to rupture of the heart was sometimes observed (Fig 2B). Liver was regularly pale or yellowish and the kidney and spleen swollen, although in some fish a very small spleen was reported. Serohaemorrhagic ascites was a common finding. The gastrointestinal tract was usually empty.

### Histopathological findings

Histological lesions as described below were found in the 80 clinically diseased fish examined. In addition 23 of the 33 apparently healthy fish from affected fish groups had similar findings although to a more moderate degree. Main organs affected were heart, red skeletal musculature and liver, but only 19% of the 103 fish had lesions in all three organs.

#### Heart

**Fig 3:**
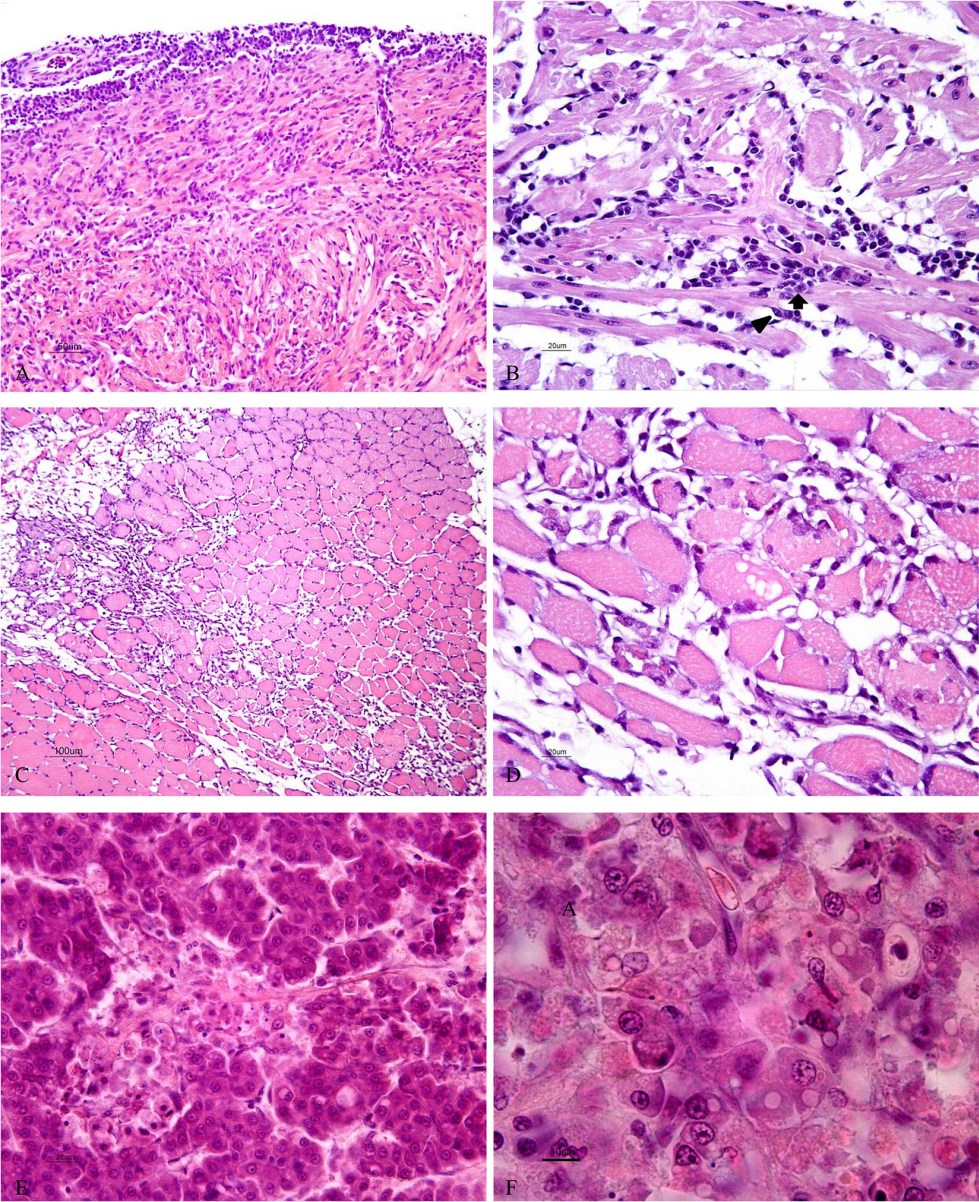
Light microscopic images of histopathology in diseased rainbow trout (H&E). (A) Heart showing pancarditis with severe epicarditis and increased cellularity in the compact and spongious layer of the heart ventricle. (B) Heart with inflammation in the spongy layer of the ventricle. The dominating cell reaction is neutrophil-like cells, both mono and polymorph nuclear (arrow) cells are seen along with hypertrophy of endocardial cells (arrow head). (C) Muscle with degeneration and necrosis of red muscle fibers and inflammatory cell infiltration, which partly has replaced the muscle fibers. (D) A close up of affected red muscle in Part C. (E) Liver with focal necrosis of hepatocytes. (F) Liver with hepatocytes at different stages of degeneration and necrosis.

The consistent organ affected was the heart. All 103 fish with histopathological findings suffered from pancarditis and for 34 fish this was the only histopathological finding (Fig 3A and 3B). Increased cellularity was seen in all parts of the heart, but often the spongious layer of the ventricle was most severely affected. Mild to severe endo-and myocarditis was accompanied by a similar degree of epicarditis. The epicardial cellularity comprised mono- and polymorph nucleated cells.

In mild cases the inflammatory response in the heart was focally to multifocally distributed, whereas severely affected hearts had an extensive and generalized reaction (Fig 3A and 3B). The cellular reaction seen in the spongious layer of both ventricle and atrium comprised circulatory, subendocardial and intramuscular neutrophils and monocytes/macrophages. Endocardial hypertrophy and proliferation were also seen (Fig 3B). In moderate and severe cases cardiomyocyte degeneration was evident and particularly in the spongious ventricle, multiple, strong eosinophilic, hyalinic cardiomyocytes, consistent with necrosis, were present. In mild cases only minor, if any, increased cellularity was found in the compact, outer muscle layer of the ventricle. When present, cellular infiltrates were localized perivascularly to the coronary vessels and in more advanced cases the cellularity was also distributed into the myocardium. Cardiomyocyte necrosis in the compact layer was also observed.

#### Skeletal musculature

Lesions in red skeletal muscle were seen in 59 of the 103 fish with heart inflammation (Fig 3C and 3D). Fish with more severely affected hearts also had more severe red muscle lesions, although red muscle was not affected in all fish with severe pancarditis. Focal, multifocal to diffuse distribution of degenerated and necrotic red fibers often infiltrated in sarcoplasm by macrophages along with endomysial cell infiltration and proliferation, were observed (Fig 3D). In severe cases the red fibers were to a large extent replaced by hypercellularity including fibrosis. Evidence of regeneration of red muscle fibers was seen in a few fish. Fifteen fish had degeneration of white muscle fibers. These findings were usually moderate and affected the dorsal white fibers below the red muscle layer and were only observed when the red muscle was severely affected.

#### Liver

Liver was affected in 35 fish, all with clinical disease signs and mild to moderate and more severe heart inflammation. Acute, focal to multifocal and partly confluating vacuolization and necrosis of hepatocytes were observed (Fig 3E and 3F). The degenerative and necrotic changes seemed to travel along sinusoids. In severe cases liver tissue was sometimes massively affected with lesions covering large areas. Subacute to chronic stages were recognized by the infiltration of inflammatory cells and mobilization of fibroblasts.

#### Other organs

In spleen and kidney increased numbers of circulatory neutrophils and monocytes were observed. Sometimes also pigmented macrophages could be seen indicative of haemosiderosis. No specific histopathological findings could be observed in other organs studied (gut, pancreas, gills).

### Haematology

Haematocrit (hcr) was measured in 65 fish from farms A, B and C. The mean hcr for the 17 fish with no histopathological findings was 40% (± 15) and for the 48 fish with typical findings 22% (± 13). The difference between the two groups was highly significant (p<0.001). About one third of the fish with typical histopathology had an hcr of 15% or below.

### Electron microscopy

High amounts of inflammatory cells were present both in the tissue and lumen of the heart, but no virus particles were detected neither in the myocytes, blood cells nor inflammatory cells.

### Bacteriology, immunohistochemistry, real time RT-PCR for known viruses and virus cultivation

No specific bacteria were isolated and *Flavobacterium psychrophilum* antigen was not detected in any of the samples tested by immunohistochemistry.

Low amounts of IPNV (Ct 33 to 35) were found at farm A in October 2013. PRV, ISAV, SAV, PMCV, VHSV or IHNV were not detected at any of the farms when examined by specific rRT-PCRs. Attempts to cultivate virus from diseased fish from farm C were unsuccessful in all tested cell lines.

### Phylogenetic tree and sequence analysis

**Fig 4:**
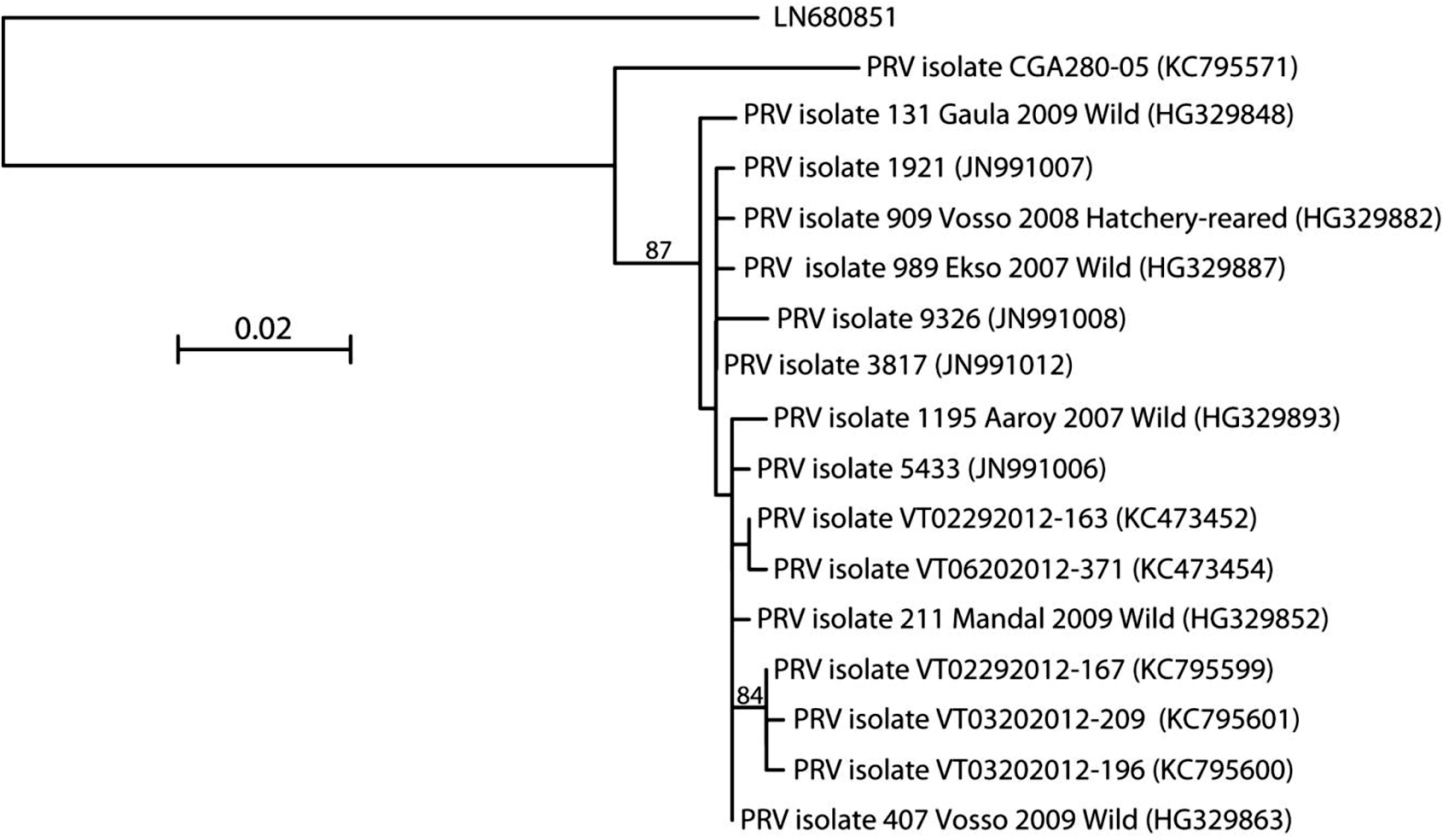
Phylogenetic tree. The phylogenetic tree is showing the genetic distance between the PRV-related virus (Acc. No. LN680851) and various piscine orthoreovirus (PRV) isolates. A neighbor-joining analysis was done using Kimura 2-parameter distances and GenBank accession numbers have been indicated. Bootstrap values above 80% are reported.

The 562 bp nucleotide sequence from the virus showed 85% identity to parts of S1 segment of PRV (Acc. No. GU994022). Both on amino acid and on nucleotide level, it was impossible to produce a reliable alignment with any other set of sequences than those originating from PRV isolates. This shows that PRV is the only known close relative to the newly detected virus, albeit the genetic distance between the newly detected virus and PRV is significantly longer than the distance between different PRV isolates (Fig 4).

### Screening of the farms for the PRV-related virus

The new rRT-PCR assay demonstrated efficient amplification and detection of the virus. No detectable products were obtained when the assay was tested against RNA derived from other pathogenic viruses. As a constant amount of nucleic acid was used in our analyses and the PCR efficiency of the rRT-PCR assay was 99.7% (data not shown), the Ct values provide an indication of the virus amount in each sample. For the purpose of this study we have correlated the Ct values and amounts of virus as follows: Ct below 25 high amounts, Ct 25–30 medium amounts and Ct over 31 low amounts of virus.

The virus was detected at all seven farms. At farms A, B, C, D and E where clinical signs and mortalities were observed, high amounts of virus were detected in moribund and dead fish. The mean Ct-value was 22 ± 5 (n = 48). Fish in chronic stage (poor performers) had medium or low amounts (mean Ct-value 33 ± 4, n = 9) or no virus at all. The difference between the two groups was highly significant (p<0.0001). Healthy fish from tanks or cages with no clinical disease had no virus, whereas apparently healthy fish from diseased groups had high amounts of virus. This was the case whether these fish had typical pathological findings (mean Ct-value 23 ± 6, n = 18) or no pathology was observed (mean Ct-value 19 ± 5, n = 11). In one sampling from farm A no virus sequence was amplified from nine clinically healthy fish with typical histopathology.

At the broodfish farms (Farms 1 & 2) no disease was observed and 3/20 and 11/46 fish respectively, were positive with low amounts of virus.

The rRT-PCR results from all seven farms were confirmed by the conventional PCR and sequencing. The obtained gene sequences showed 100% identity to each other.

## Discussion

This is the first description of a new disease in rainbow trout observed in three hatcheries on the west coast of Norway in 2013–14. All three fresh water farms received eggs and/or fish from the same broodstock source. A PRV-related virus was detected at all the affected sites, as well as in the broodstock. Moreover, at two marine sites, apparently healthy fish from affected hatcheries developed the disease after sea transfer. Investigations led to detection of the PRV-like virus also at the affected marine sites.

The disease described is characterized by inflammation of the heart and red skeletal muscle, liver necrosis, anaemia and moderate to high mortality rates. With exception of anaemia, the pathological findings resemble HSMI, a common disease in Atlantic salmon in Norway. HSMI has not been described in rainbow trout or other fish species. Although cases of HSMI in salmon fresh water production are reported, the major impact of this disease seems to occur 5–9 months after sea transfer. In the present study the disease occurred primarily in young fish in the fresh water stage. Despite similar pathology, the course of the new disease seems to be quite different compared to HSMI.

Heart inflammation in rainbow trout, similar to what is described in the present paper, has been described by MacWilliams and co-workers [3], in a few fish used in an infection trial with ISAV. The main finding was epicarditis which subsequently seemed to affect the compact myocardium in severely affected fish. This is in contrast to our findings, where the spongious layer seemed to be most severely affected. Additionally, in their case no reaction in the red muscle was noted and liver necrosis was seen in only a few fish. The cause of the inflammatory reaction in the ISAV trial was unclear. In our case all fish tested for ISAV were negative.

Different observations suggest that the disease seen in rainbow trout is most likely infectious. It seemed to spread both between tanks and between individuals within a tank. Clinical signs preceded the mortality, and the most prominent pathological finding is inflammation in several organs, indicative of septicaemia. Liver necrosis is often seen in fish with circulatory failure and is possibly a secondary feature due to heart failure. In this case the degenerative changes seemed to travel along the sinusoids, which also may indicate a haematogenous spread of an agent. Neutrophils were involved in the inflammation process which could point to a bacteriological etiology, but no bacteria were detected, neither by culture nor by special staining of histological sections examined by light microscopy. As *Flavobacterium psychrophilum* infections in rainbow trout may cause an inflammatory reaction in the heart in chronic cases, specific tests for this bacterium were performed, with negative results. Still, bacteria, as a possible cause, cannot be fully excluded. Neither parasites nor fungi were found and the inflammation characteristics showed no classical signs of infections caused by such agents.

Because the pathological findings resembled HSMI, which is associated with PRV, we looked for a PRV-like virus in the samples. And, indeed, the detected virus showed 85% identity with PRV in the part of the investigated genome. The genome of reoviruses is segmented and we have so far only detected a partial sequence corresponding to one of the segments. The virus may possibly be a new variant of PRV rather than a new species, but full genome characterization is necessary to confirm classification within the family *Reoviridae*, genus affiliation and the relationship to PRV.

We did not find any virus particles in the electron microscopic studies of hearts and blood cells in which high amounts of the PRV-like virus have been detected by rRT-PCR. Also in earlier studies of HSMI in Atlantic salmon, virus particles were difficult to detect in inflamed tissues [24], [25]. Large amounts of PRV-particles have later been shown in the red blood cells by TEM examinations of blood from early stages of PRV infections from challenge experiments [26]. Material from early stages of the infection might also be necessary to reveal the virus particles, and challenge trials should provide optimal material for future TEM studies.

A relatively high proportion of the diseased fish in the present study suffered from anaemia. Anaemia in salmonids, including rainbow trout, has previously been linked to erythrocytic inclusion body syndrome (EIBS) [27]. Electron microscopic images of the EIBS-inclusions reveal virus particles that resemble PRV [28], [26]. To our knowledge it has not been confirmed whether all or some of the cases of EIBS are related to PRV or other reoviruses, and this needs further investigations. Our TEM studies did not reveal any viral inclusions in the red blood cells despite positive PCR detections with low CT-values of the PRV-like virus in blood. Investigation of blood at an earlier stage of the infection could be essential to reveal whether the newly detected virus can be related to EIBS.

An association between the disease and the virus is supported by findings of high amounts of virus in clinically diseased fish at the acute and subacute-chronic stage. Low viral loads or in some cases no virus at all, were detected in fish in late stages of the disease, which is expected when the immune system manages to fight the virus. Moreover, at the affected farms, large amounts of the virus were detected in some apparently healthy fish within diseased fish groups. The presence of high amount of virus before onset of clinical disease is a typical observation for diseases involving viral agents. However, during our investigations, one of the samplings consisting of fish with typical pathological findings from farm A was negative for virus when examined by real time RT-PCR. Although one would expect those to be positive assuming that there is an association between the pathological changes and the virus, this observation could be explained by undetectable viral loads. On the other side, this may also indicate another cause of the disease than the PRV-related virus. Nevertheless, the fact that the disease display similarities with HSMI in salmon and the virus resembles PRV, can point to a link between disease and virus also in the rainbow trout.

Controlled challenge trials are necessary to reveal the impact of the novel virus on fish health, establish the true cause of the new disease and describe the pathogenesis. Challenge trials with uncultivable viruses pose a challenge as filtered tissue from diseased fish must be used. This leaves a possibility that more than one infectious agent is present in the inoculum, and complicates interpretation of the results. New cell lines or other strategies of cultivating fish viruses are needed to enhance investigations of both the newly detected virus and other viruses for which cultivation methods are still lacking.

Further studies to reveal the pathogenesis and tissue distribution of the PRV-like virus at different stages of the infection are needed. The tissue distribution at the acute and chronic stage of the infection may vary considerably. Blood was used for detection of the virus in the clinically healthy broodfish. The prevalence and amount of virus in these fish were low pointing to a possible carrier state. Longitudinal studies will be useful to reveal how long infected fish might harbor the virus and in which organs it accumulates.

The fact that all diseased fish originated from the same broodfish source indicates a possible vertical component in the spread of the disease that needs to be further investigated.

The spread of the disease described here points towards an infectious etiology. This disease is either triggered by the introduction of a new infectious agent, or by a change in the virulence of an already established agent. Diseases are often multifactorial, so a number of different factors including general health status of the fish and water quality may have contributed to the disease outbreaks. Whether the virus detected at the affected rainbow trout farms can be associated with the new disease or other health issues need further investigations. If so, it is of major importance to reveal the reservoir of the PRV-related virus and stop the spread into new areas. The PCR methods established in this study will be useful to map present distribution of the virus and examine historical material in order to reveal earlier distributions. Challenge trials are necessary to investigate the transmissibility, virulence and pathogenesis of the PRV-like virus both in rainbow trout and other possible susceptible species.
